# Supplementary material for: A method to estimate absolute odorant concentration of olfactory stimuli
Source: PLoS One. 2026 Jan 2;21(1):e0337336. doi: 10.1371/journal.pone.0337336 (PMC12758820; doi:10.1371/journal.pone.0337336)
Supplement: S1 Table — Publications where authors have tentatively or conclusively identified GLVs in the ambient air of the studied ecosystem, within 35 m above the vegetation canopy. We extracted the estimates of average, median, maximum or range of GLV concentrations. Note that the reported values always represent an average over a certain volume of sampled air. The PTR-MS detects and quantifies the products of a gas sample’s ionization, classified by their mass to charge ratio (m/z). When a complex sample such as ambient air is analyzed, it is not possible to discriminate among individual compounds with the same m/z. Identification of atmosphere components by PTR-MS therefore remain tentative unless further analyses confirm which VPCs actually contribute to the detected ions. Ionization of hexenyl acetate produces notably m/z 83 (abundant but unspecific) and m/z 143 (specific but much less abundant [1]). Several other GLVs generate mostly m/z 83 when ionized. This table summarizes the reported concentrations of (A) PTR-MS ions to which Z3HA, (B) PTR-MS ions that are markers of other GLVs, and (C) GLV concentrations estimated by other methods. LOD = limit of detection, sd = standard deviation, ppbv = parts per billion by volume. (DOCX) [file pone.0337336.s004.docx]

**Table S1A PTR-MS data: m/z 83 and 143**

| Reference | Ecological context | ion | author's identification | Reported concentrations (ppbv) | | notes |
| --- | --- | --- | --- | --- | --- | --- |
| ^[2]^ | Creosote (*Larrea tridentata*) bushland, Arizona  summer, 3m above canopy | m/z 83 | 3-methyl furan, hexanal and 3-hexenyl acetate | 0.1-0.15 | range, noon time | Contributor compounds confirmed by GC-PTR-MS and GC-MS on cresoste branch headspace extracts |
|  |  |  |  | 0.01-0.1 | range, 48h time series |  |
|  |  | m/z 143 | nonanal, 3-hexenyl acetate, 1-chloro-2-methoxy benzene | 0.6-1 | range, noon time |  |
|  |  |  |  | 0-0.6 | range, 48h time series |  |
| ^[3]^ | *Pseudotsuga menziesii* forest, Denmark  summer, under, in and up to 13m above canopy | m/z 83 | hexenols, hexanal (E,Z)-2-hexenyl acetate | 0,4 | above canopy average | LOD 0.6-0.7 ppbv |
|  |  |  |  | 0.18-0.34 | averages per time of day and height in canopy |  |
| ^[4,5]^ | *Citrus sinensis* orchard, California  year round, in and up to 5m above canopy | m/z 83 | hexanal, hexenols | 0.14, 0.25, 0.15 | midday averages during winter, flowering and summer | LOD 0.02 ppbv  occasional peaks up to 4ppbv during flowering |
|  |  | m/z 83.086^*^ | hexanal, hexenols | 0.07, 0.17 | day and night time averages in summer | m/z 143.114 (=hexenyl acetate) not in the list of detected ions |
| ^[6]^ | Palm oil plantation, Borneo april-july 3m above canopy | m/z 83 | hexanal | 0,5 | global average |  |
|  |  |  |  | 1 ± 0.5 | midday average ± sd |  |
| ^[6]^ | Rainforest Borneo  april to july 35 m above canopy | m/z 83 | hexanal | very low |  |  |
| ^[7]^ | Rainforest Borneo  april to july, 35 m above canopy | m/z 83 | hexanal, hexenols | 0.06±0.02 | global mean and variance | LOD 0.04 ppbv |
|  |  |  |  | 0.06, 0.04-0.09 | midday average and range |  |
|  |  |  |  | 0.04, 0.02-0.06 | night time average and range |  |
| ^[8]^ | Permanent managed grassland, Swizerland  summer 1.2m above ground  during and after grass cutting/hay removal | m/z 83 | (Z)-3-hexen-1-ol, (E)-3-hexenol, (E)-2-hexenol, hexanal, (E,Z)-3-hexenyl acetate | 0.92, 0.19, 0.22, 0.05 | day time average, 1 day, 2 days, 3 days and 4 days after grass cutting | day 4 concentration comparable to pre-cutting  (Z)-3-hexenyl acetate represents 60-90% of m/z 83 from dominant grass species emissions (GC-PTR-MS on headspace) |
| ^[9]^ | mature rainforest, Amazonia  during a drought event, under, in and up to 10 m above canopy | m/z 83 | 3-methyl furan, hexenols | 0.05-0.5, 0.2-0.8 | monthly averages, mid-canopy and above canopy | Identity of contributor compounds confirmed by GC-PTR-MS on above canopy air. Undetectable at ground level |
| ^[10]^ | Urban lawn, Austria  before, during and after mowing | m/z 83 | hexanal, hexenols | up to 2-6 | peaks after mower passage | Very low concentration before mowing  m/z 143 not detected |
|  |  |  |  | 0-0.7 | 3 to 8h post mowing |  |
| ^[11]^ | Oak-dominated forest in agricultural landscape, Italy  summer, 4m above canopy | m/z 83.086^*^ | fragment of C6 GLVs | 0.8 | whole campaign average | m/z 101.0961 (parent ion for hexanal and hexenols) is detected.  m/z 143.114 (parent ion for hexenyl acetate) is not. |

**Table S1B PTR-MS data: other GLV characteristic ions**

| Reference | Ecological context | ion | Author’s identification | Concentrations (ppbv) | | notes |
| --- | --- | --- | --- | --- | --- | --- |
| ^[4]^ | Citrus sinensis orchard, California  year round, in and up to 5m above canopy | m/z 99 | hexenal | 0.03, 0.04, 0.06 | midday averages during winter, flowering and summer | LOD 0.14 ppbv  occasional peaks up to 4ppbv during flowering season |
| ^[5]^ |  | m/z 99.078^*^ | hexenal | 0.04, 0.05 | day and light time averages in summer |  |
| ^[6]^ | Rainforest Borneo  april to july, 35 m above canopy | m/z 85 | hexenols | very low |  |  |
|  | Palm oil plantation, Borneo  3 m above canopy | m/z 85 | hexenols | very low |  |  |
| ^[8]^ | Permanent managed grassland on Swiss plateau  summer 1.2m above ground  during and after grass cutting/hay removal | m/z 81 | (E)-2-hexenal, (Z)-3-hexenal, pinene fragments | 0.82, 0.16, 0.17, 0.05 | day time average, 1 day, 2 days, 3 days and 4 days after grass cutting | day 4 concentration comparable to pre-cutting (Z)-3-hexenal represents 100% of m/z 81 from most dominant grass species emissions (GC-PTR-MS on headspace) |
| ^[9]^ | mature rainforest, Amazonia  during a drought event, under, in and up to 10m above canopy | m/z 85 | hexanol | 0.04-0.16 | monthly average mid canopy and above canopy | Undetectable at ground level. GC-PTR-MS analysis of above canopy air reveals only 1 peak |
| ^[10]^ | Urban lawn, Austria  before, during and after mowing | m/z 81 | hexenal | <0.1 | before mowing |  |
|  |  |  |  | 2-6 | peaks post mower passage |  |
|  |  |  |  | 0-0.7 | 3 to 8h post mowing |  |
| ^[12,13]^ | Boreal forest dominated by *Pinus sylvestris*, Finland  year round, in canopy | m/z 101 | hexanal, (Z)-3-hexen-1-ol | 0.22-2.11 | monthly medians | LOD 0.22 |
|  |  | m/z 99 | hexenals | 0.05 | global median |  |

**Table S1C GLV concentrations estimated by other methods**

| Reference | Ecological context | technique | Compounds | Concentrations (ppbv) | |
| --- | --- | --- | --- | --- | --- |
| ^[14,15]^ | Spruce (*Picea abies*) forest, Bavaria  summer, in and 5 m above canopy | HPLC | trans-2-hexenal | 0.05-0.55 | whole campaign ranges |
|  |  |  | nonanal | 0-0.45 |  |
|  |  |  | hexanal | 0-0.28 |  |
|  |  |  | heptanal | 0-0.15 |  |
|  |  |  | octanal | 0.0.25 |  |
|  |  |  | decanal | 0-0.02 |  |
| ^[16]^ | Hemiboreal forest, dominated by *Picea abies*, Estonia  year round, under and in canopy  by clear sky weather only | GC-MS,  headspace | (Z)-3-hexen-1-ol | 0.001-1 | averages per month and height |
|  |  |  | 1-hexanol | 0.0005-1.3 | averages per month and height |

* measured by PTR-ToF-MS. This instrument measures mass to charge ratio with enough precision to deduce the molecular formula of the detected ion.
