# Supplementary material for: A method to estimate absolute odorant concentration of olfactory stimuli
Source: PLoS One. 2026 Jan 2;21(1):e0337336. doi: 10.1371/journal.pone.0337336 (PMC12758820; doi:10.1371/journal.pone.0337336)
Supplement: S1 File — (DOCX) [file pone.0337336.s005.docx]

# Supporting Information

# Model of odorant release from a liquid to a headspace compartment

We consider a system composed of a liquid and a headspace compartment, separated by an interface, the liquid’s surface (Figure S1).

Hypothesis 1: The bulk liquid and bulk headspace compartments are perfectly stirred, i.e. odorant concentration is always uniform across their whole volume: noted $C_{l}$ and $C_{h}$. This is at the exception of two thin boundary layers on each side of the interface.

Hypothesis 2: Transport across the interface itself is instantaneous, so the two interfacial layers are always at thermodynamic equilibrium with one another. At any moment:

$C_{h}^{*}=K_{hl}*C_{l}^{*}$ (A1)

Mass fluxes from bulk liquid to liquid interfacial layer J_l->l*_ and from headspace interfacial layer to bulk headspace $J_{h^{*}\to h}$ (mol m^-^² s^-1^) depend on the difference between bulk and interfacial concentrations and on transfer coefficients k_l_ and k_h_ (m/s):

$J_{l\to l*}=k_{l}*\left( C_{l}-C_{l}^{*} \right)$ (A2)

$J_{h^{*}\to h}=k_{h}*\left( C_{h}^{*}-C_{h} \right)$ (A3)

If multiplied by K_hl_/k_l_, equation (A2) becomes

$J_{l\to l*}*\frac{K_{hl}}{k_{l}}=K_{hl}*\left( C_{l}-C_{l}^{*} \right)$ (A4)

When incorporating equation (A1) and dividing by k_h_, equation (A3) becomes

$J_{h*\to h}*\frac{1}{k_{h}}=K_{hl}*C_{l}^{*}-C_{h}$ (A5)

Hypothesis 3: No accumulation occurs within the interfacial layers. The mass flow into the liquid interfacial layer is equal to the mass flow out from the headspace interfacial layer, and equal to J_l->h_ the global mass flow through the interface from bulk liquid to bulk headspace:

$J_{l\to l*}=J_{h*\to h}=J_{l\to h}$ (A6)

This allows to deduce the value of J_l->h_ : summing up equations (A4) and (A5) and incorporating equation (A6) we get

$$J_{l\to h}*\frac{K_{hl}}{k_{l}}+J_{l\to h}*\frac{1}{k_{l}}=K_{hl}*\left( C_{l}-C_{l}^{*} \right)+K_{hl}*C_{l}^{*}-C_{h}$$

which simplifies to

$$J_{l\to h}*\left( \frac{1}{k_{h}}+\frac{K_{hl}}{k_{l}} \right)=K_{hl}*C_{l}-C_{h}$$

and further to

$J_{l\to h}=k_{glob}*\left( K_{hl}*C_{l}-C_{h} \right)$ (A7)

where k_glob_ is the global mass transfer coefficient, defined as

$\frac{1}{k_{glob}}=\frac{1}{k_{h}}+\frac{K_{hl}}{k_{l}}$ (A8)

*Dynamics of odorant concentration inside the source*

From equation (A7), we can deduce a set of differential equations describing how odorant concentrations in liquid and in headspace evolve over time, as a function of mass gains from the preceding compartment and mass losses to the following compartment.

Changes in C_l_ are only the result of loss of molecules through the interface:

$\frac{{dC}_{l}}{dt}=\frac{{-J}_{l\to h}*A}{V_{l}}$ (A9)

where A is the area of the interface (m²) and V_l_ the volume of the liquid compartment (m^3^).

On the contrary, changes in C_h_ result from mass gain through the interface and mass loss due to headspace dilution by the airflow running through the source, Q_s_ (m^3^.s^-1^):

$\frac{{dC}_{h}}{dt}=\frac{J_{l\to h}*A-Q_{s}*C_{h}}{V_{h}}$ (A10)

where V_h_ is the volume of the headspace compartment. Q_s_ carries the odorized air through the outlet of the source, and the concentration delivered by the source can be considered equal to C_h_ whenever Q_s_ > 0.

*Mass transport to the preparation*

The outlet of the source is connected to the transport compartment, which is focused on the insect’s antenna under study. Following the same principle as above, odorant concentration inside the transport compartment, C_c_, results from mass gain from source headspace and mass loss due to dilution by the carrier airflow:

$\frac{{dC}_{c}}{dt}=\frac{Q_{s}*C_{h}-Q_{c}*C_{c}}{V_{c}}$ (A11)

Odorant concentration delivered on the preparation can be considered as equal to C_c_ at any time assuming the transport compartment is well mixed due to mixing of 8 air streams at its entry.

*Nondimensionalization*

Sources prepared with odorants having very different K_hl_ values and/or using solutions of very different $C_{l}^{0}$ values lead to concentrations varying by many orders of magnitude. For comparisons between compounds with very different K_hl_ values, it is convenient to transform these values into variables ranging between 0 and 1. This is done by nondimensionalization, i.e. by dividing all concentrations by $C_{l}^{0}$ in the liquid phase and $C_{h}^{0}$ in the air, respectively:

$Y_{l}=\frac{C_{l}}{C_{l}^{0}} ; Y_{h}=\frac{C_{h}}{C_{h}^{0}} ; Y_{c}=\frac{C_{c}}{C_{h}^{0}}$ (A12)

where Y_l_, Y_h_ and Y_c_ represent respectively the dimensionless liquid, headspace and transport compartment concentrations.

Once nondimensionalized, the differential equations describing the time course of odorant concentration inside each section of the odor delivery device become:

$\frac{{dY}_{l}}{dt}=\frac{k_{glob}*A}{V_{l}}*K_{hl}*\left( Y_{h}-Y_{l} \right)$ (A13)

$\frac{{dY}_{h}}{dt}=\frac{k_{glob}*A}{V_{h}}*\left( Y_{l}-Y_{h} \right)-\frac{Q_{s}*Y_{h}}{V_{h}}$ (A14)

$\frac{{dY}_{c}}{dt}=\frac{Q_{s}*Y_{h}-Q_{c}*Y_{c}}{V_{c}}$ (A15)

**References**

^[1]^ Fall R (1999) Volatile organic compounds emitted after leaf wounding: On-line analysis by proton-transfer-reaction mass spectrometry*.* *J Geophys Res Atm* 104(D13):15,963-15,974. DOI: 10.1029/1999JD900144

^[2]^ Jardine K, Abrell L, Kurc SA, Huxman T, Ortega J, Guenther A (2010) Volatile organic compound emissions from *Larrea tridentata* (creosotebush)*.* *Atmos. Chem. Phys.* 10:12191–12206. DOI: 10.5194/acp-10-12191-2010

^[3]^ Copeland N, Cape JN, Nemitz E, Heal MR (2014) Volatile organic compound speciation above and within a Douglas fir forest*.* *Atmos Environ* 94:86-95. DOI: 10.1016/j.atmosenv.2014.04.035

^[4]^ Fares S, Park JH, Gentner DR, Weber R, Ormeño E, Karlik J, Goldstein AH (2012) Seasonal cycles of biogenic volatile organic compound fluxes and concentrations in a California *citrus* orchard*.* *Atmos. Chem. Phys.* 12:9865–9880. DOI: 10.5194/acp-12-9865-2012

^[5]^ Park JH, Goldstein AH, Timkovsky J, Fares S, Weber R, Karlik J, Holzinger R (2013) Eddy covariance emission and deposition flux measurements using proton transfer reaction – time of flight – mass spectrometry (PTR-TOF-MS): comparison with PTR-MS measured vertical gradients and fluxes*.* *Atmos. Chem. Phys.* 13:1439–1456. DOI: 10.5194/acp-13-1439-2013

^[6]^ MacKenzie AR, Langford B, Pugh TA, Robinson N, Misztal PK, Heard DE, Lee JD, Lewis AC, Jones CE, Hopkins JR, Phillips G, Monks PS, Karunaharan A, Hornsby KE, Nicolas-Perea V, Coe H, Gabey AM, Gallagher MW, Whalley LK, Edwards PM, Evans MJ, Stone D, Ingham T, Commane R, Furneaux KL, McQuaid JB, Nemitz E, Seng YK, Fowler D, Pyle JA, Hewitt CN (2011) The atmospheric chemistry of trace gases and particulate matter emitted by different land uses in Borneo*.* *Philos Trans R Soc Lond B Biol Sci* 366(1582):3177-3195. DOI: 10.1098/rstb.2011.0053

^[7]^ Langford B, Misztal PK, Nemitz E, Davison B, Helfter C, Pugh TAM, MacKenzie AR, Lim SF, Hewitt CN (2010) Fluxes and concentrations of volatile organic compounds from a South-East Asian tropical rainforest*.* *Atmos. Chem. Phys.* 10(17):8391-8412. DOI: 10.5194/acp-10-8391-2010

^[8]^ Davison B, Brunner A, Ammann C, Spirig C, Jocher M, Neftel A (2007) Cut-induced VOC emissions from agricultural grasslands*.* *Plant Biol* 9:e60-e68. DOI: 10.1055/zs-2007-965043

^[9]^ Jardine KJ, Chambers JQ, Holm J, Jardine AB, Fontes CG, Zorzanelli RF, Meyers KT, de Souza VF, Garcia S, Gimenez BO, Piva LR, Higuchi N, Artaxo P, Martin S, Manzi AO (2015) Green leaf volatile emissions during high temperature and drought stress in a central Amazon rainforest*.* *Plants* 4(3):678-690. DOI: 10.3390/plants4030678

^[10]^ Karl T, Fall R, Jordan A, Lindinger W (2001) On-line analysis of reactive VOCs from urban lawn mowing*.* *Environ. Sci. Technol.* 35(14):2926-2931. DOI: 10.1021/es010637y

^[11]^ Schallhart S, Rantala P, Nemitz E, Taipale D, Tillmann R, Mentel TF, Loubet B, Gerosa G, Finco A, Rinne J, Ruuskanen TM (2016) Characterization of total ecosystem-scale biogenic VOC exchange at a Mediterranean oak–hornbeam forest*.* *Atmos. Chem. Phys.* 16(11):7171-7194. DOI: 10.5194/acp-16-7171-2016

^[12]^ Ruuskanen TM, Taipale R, Rinne J, Kajos MK, Hakola H, Kulmala M (2009) Quantitative long-term measurements of VOC concentrations by PTR-MS: annual cycle at a boreal forest site*.* *Atmos. Chem. Phys. Discuss.* 9(1):81-134. DOI: 10.5194/acpd-9-81-2009

^[13]^ Patokoski J, Ruuskanen TM, Hellen H, Taipale R, Gronholm T, Kajos MK, Petaja T, Hakola H, Kulmala M, Rinne J (2014) Winter to spring transition and diurnal variation of VOCs in Finland at an urban background site and a rural site*.* *Boreal Environ. Res.* 19(2):79-103. DOI: 10138/165174

^[14]^ Klemm O, Held A, Forkel R, Gasche R, Kanter H-J, Rappenglück B, Steinbrecher R, Müller K, Plewka A, Cojocariu C, Kreuzwieser J, Valverde-Canossa J, Schuster G, Moortgat GK, Graus M, Hansel A (2006) Experiments on forest/atmosphere exchange: Climatology and fluxes during two summer campaigns in NE Bavaria*.* *Atmos. Environ.* 40:3-20. DOI: 10.1016/j.atmosenv.2006.01.060

^[15]^ Müller K, Haferkorn S, Grabmer W, Wisthaler A, Hansel A, Kreuzwieser J, Cojocariu C, Rennenberg H, Herrmann H (2006) Biogenic carbonyl compounds within and above a coniferous forest in Germany*.* *Atmos. Environ.* 40:81-91. DOI: 10.1016/j.atmosenv.2005.10.070

^[16]^ Noe SM, Hüve K, Niinemets Ü, Copolovici L (2012) Seasonal variation in vertical volatile compounds air concentrations within a remote hemiboreal mixed forest*.* *Atmos. Chem. Phys.* 12(9):3909-3926. DOI: 10.5194/acp-12-3909-2012
