# Supplementary material for: A method to estimate absolute odorant concentration of olfactory stimuli
Source: PLoS One. 2026 Jan 2;21(1):e0337336. doi: 10.1371/journal.pone.0337336 (PMC12758820; doi:10.1371/journal.pone.0337336)
Supplement: Attachment — Submitted filename: Response to reviewers.pdf [file pone.0337336.s007.pdf]

The manuscript is well written in its current form.

We thank the reviewer for this comment and address the following ones point by point below.

The authors should take care of the minor suggestions to correct few mistakes:

1. Line 163 -function geeglm() of package geepack in R). Is this an error geeglm() in this line? please correct or specify.
2. Line 265-compartments over time were fed into the differential equation solver function ode() from package. What is ode()?

geeglm() and ode() are standard functions within the R software environment for data analysis and model computation. The sentences are accurate as stated. We believe that most readers will already be familiar with the specific coding syntax employed by the software R. Providing a detailed explanation of R syntax may go beyond the scope of the manuscript. For these reasons, we propose to leave these sentences unchanged.

3. The authors have discussed mineral and paraffin oil in the discussion. What about DMSO that many labs use to dissolve odorants?

To our knowledge, DMSO has only been used to dissolve odorants in aqueous solutions (e.g. when working with olfactory receptors expressed in Xenopus oocytes or with olfactory receptor neurons in culture). However, the model we describe is applicable to any solvent, provided that the partition coefficients are determined for each solute–solvent pair. We made this point clear by adding "and solvent" in the manuscript (line 671).

4. Use the same nomenclature for (Z)-3-hexen-1-ol everywhere in the manuscript. Check other chemical names as well.

This has been corrected in line 121 of the main text and in Table S1A.

5. Line 837-Table 1 - List of variables and of parameters of the model. Rewrite this as " List of variables and parameters of the model".

This mistake has been corrected.

6. Line 849- kglob mass transfer coefficient (m.s-1). Make m capital as "M" of mass to keep uniformity with other abbreviations.

This mistake has been corrected.

Reviewer #2: This study, authored by Conchou et al., investigated the estimation of absolute odour concentration for ORN stimuli in A. ipsilon sp. The introduction and discussion sections of the manuscript are well written. However, the manuscript requires a few modifications to improve its readability.

We thank the reviewer for this comment and address the following ones point by point below.

Major comment:
1. The manuscript is well written by the authors and the English is acceptable. However, the authors need to simplify a few sentences to make them clearer for readers. For example, on line 22, 'the present model or approach'; on line 33: 'The difficulty'?
Line 62: 'Such a solution meets the requirements?'

These mistakes have been corrected.

2. Methods: Line 117: Why did the authors only select 11 VPC compounds for testing, and why did they choose Z3HA for the EAG dose-response and pheromone sensitivity background analysis (Figures 8 and 9)?

We clarified it by adding line 126: "This selection of VPCs provides a basis for demonstrating the validity and potential of our model of prediction of the odorant concentration delivered by an odor delivery system. Among the 11 selected VPCs, Z3HA was chosen for electrophysiological experiments to evaluate whether, at ecologically relevant concentrations, it can interfere with sex pheromone detection in *A. ipsilon* moths.

3. The authors elaborated on the methodology with a lot of quantification and various formulas for the stimulated system. I suggest that the authors reconsider transferring a few of the more complex formulas to the supplementary information file from the methodology section.

We have moved a substantial portion of the formulae to the Supplementary Information.

4. The supplementary files are not available for verification (S1 figures).

There is one single figure in Supplementary Information. The raw data can be downloaded until November 5, 2025 from:
https://filesender.renater.fr/?s=download&token=2ba65324-446d-4f6e-8093-dc5b0b25b6ac
However, please not that they were plotted without any specific treatment.

5. I suggest that the authors provide a conclusion at the end of the discussion rather than with the results.

The conclusion made at the end of the results was moved to the discussion, in the paragraph spanning lines 683-697.

6. On line 526, the authors state that 'linalool and β-caryophyllene showed sharper dynamics at stimulus onset and offset with the PTR-MS than with the PID'. Why do the authors consider Z3HA rather than linalool and β-caryophyllene?

We are sorry, but we do not fully understand the question here.

- Maybe 'not' is missing and it should read as: 'Why don't the authors consider Z3HA rather than linalool and β-caryophyllene?'.
  The choice of molecules tested by PTR-MS was arbitrary; we selected several compounds with different PID dynamics. If the reviewer regrets that we did not select Z3HA for PTR-MS experiments because we used it for electrophysiology, we would like to point out that we took precautions regarding the equilibration times before opening the valve that delivered the stimulus to the preparation.
- If the reviewer did not forget 'not', then the answer is that we selected Z3HA because it affects pheromone perception under laboratory conditions.

7. Figure 9: What does ppbv mean in this legend? (C) To the pheromone in the Z3HA background. Are Z3HA backgrounds expressed as both dilution in mineral oil and concentration delivered at a dynamic pseudo-stationary regime (ppbv)?

ppbv stands for parts per billion by volume. We have now defined this abbreviation at its first occurrence (line 612). We thank the reviewer for bringing this oversight to our attention.
